# A rare cause of respiratory distress in preterm infants: a case report of acquired subglottic cysts

**DOI:** 10.1186/s13052-024-01784-w

**Published:** 2024-10-16

**Authors:** Luca Barchi, Giulia Russo, Sara Donvito, Giulia Barbato, Francesco Leo, Elisa Iannella, Angelo Ghidini, Lorenzo Iughetti, Giancarlo Gargano

**Affiliations:** 1 Department of Medical and Surgical Sciences of the Mother, Children and Adults, Post-Graduate School of Paediatrics, University of Modena and Reggio Emilia, Via del Pozzo 71, Modena, 41125 Italy; 2 Department of Otolaryngology - Head and Neck Surgery, University Hospital of Modena and Reggio Emilia, Via del Pozzo 71, Modena, 41125 Italy; 3 Departement of Mother and Child, Neonatal Intensive Care Unit, Azienda USL-IRCCS di Reggio Emilia, Reggio Emilia, 42123 Italy; 4 ENT Department, Anaesthesiology Unit, Azienda USL-IRCCS di Reggio Emilia, Reggio Emilia, 42123 Italy; 5 ENT Department, Otolaryngology Unit, Azienda USL-IRCSS of Reggio Emilia, Reggio Emilia, 42123 Italy

**Keywords:** Subglottic cysts, Neonatal, Preterm infants, Dyspnea, Intubation related disease

## Abstract

**Background:**

The Subglottic Cysts (SGCs) are a rare cause of respiratory distress in infants. Typical risk factors include male gender, extreme prematurity, gastro-oesophageal reflux and invasive ventilation, the latter being associated with mucosal damage and blockage of the subglottic cysts’ ducts. We describe a case of acquired subglottic cysts in a premature infants presented with respiratory distress.

**Case presentation:**

A premature male infant was born at 25 weeks + 2 days with a history of monochorionic diamniotic twin pregnancy with twin-to-twin transfusion syndrome. During hospitalization, invasive mechanical ventilation was necessary for a total of 18 days; the patient was discharged at postmenstrual age of 40 weeks + 1 day in good condition. At 43 weeks post-menstrual age, he presented to our department with mixed stridor and worsening of respiratory dynamics. A laryngotracheoscopy evaluation was performed. The exam showed the presence of multiple SGCs causing an almost complete obstruction of the airway. Because of the significant reduction of the airway’s patency, the child underwent a tracheotomy and thereafter cysts’ removal using cold steel microinstruments. A better airway patency was restored although a slight glottic edema persisted. The histopathology confirmed the benign nature of the lesions. Successive controls showed a completely patent airway and absence of SGCs.

**Conclusion:**

In conclusion, SGCs should be considered in preterm infants with respiratory distress previously intubated, which cannot be explained by the most common causes. Early diagnosis and treatment are fundamental to reducing the morbidity and mortality associated with this disease.

## Background

Subglottic cysts (SGCs) are a rare cause of respiratory distress and upper airway obstruction in previously intubated infants [1]. Most cases present in extremely premature newborn, suffering from respiratory distress, and have been intubated for many days [2]. The estimated incidence of SGCs is 1.9 for 100,000 live births [3], but there is a steady increase in the number of cases of SGCs. This may be attributed to the increasing survival rates of preterm infants and increasing awareness of SGCs [4]. Herein, we describe a case of a premature infant with subglottic cysts treated by the cold exeresis method and tracheostomy.

## Case presentation

**Fig. 1:**
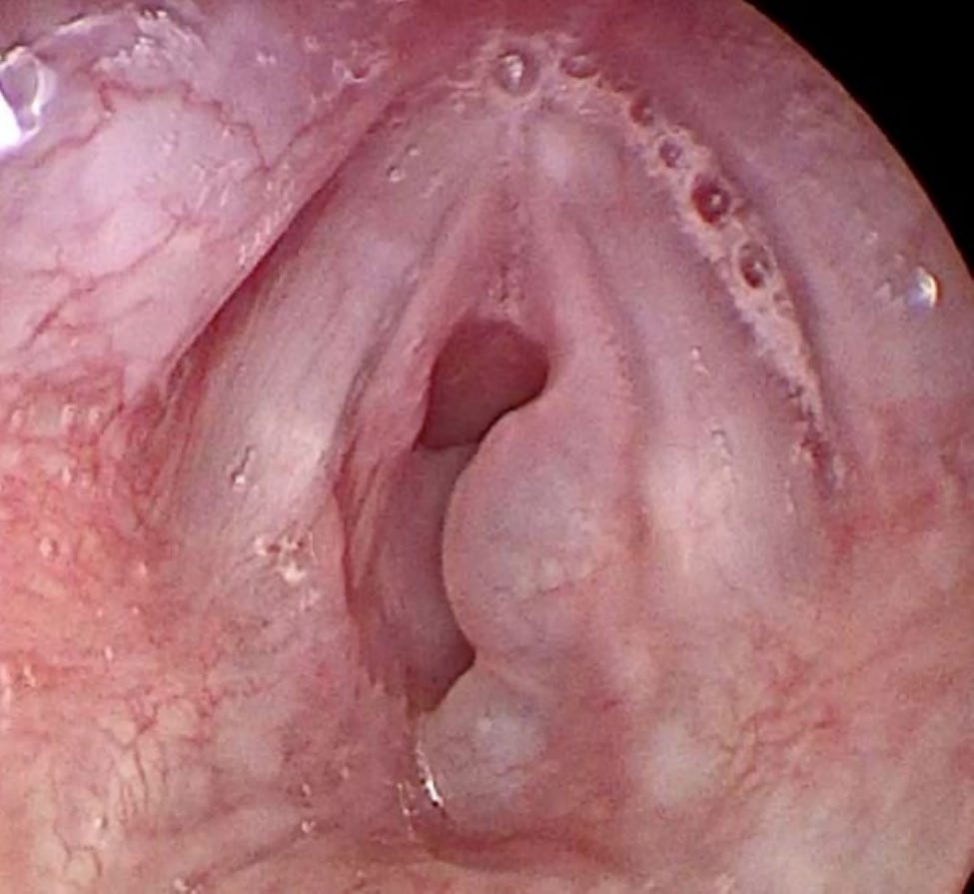
Laryngoscopy showing glottic and subglottic cysts with narrowing of the airway patency

Born at 25 weeks + 2 days gestational age by emergency section due to unstoppable labour with rupture of the membranes in a monochorionic diamniotic twin pregnancy. Pregnancy was characterised by a twin-to-twin transfusion syndrome (receving fetus). Incomplete lung maturity. At birth, there was no respiratory activity and the heart rate (HR) was below 100 bpm. Need for intubation at 4 min of life due to suboptimal oxigen saturation. The first dose of endotracheal surfactant was administered due to the persistent suboptimal saturations and HR < 100 bpm with FiO2 at 100 at 10 min of life. This resulted in a gradual improvement ofvital parameters. The patient was transferred to the Neonatal Intensive Care Unit (NICU) with mechanical invasive respiratory support. During hospitalization, the infant was supported by invasive mechanical ventilation for 18 days. Two doses of surfactant were administered at 24 h and 96 h of life. The patient was discharged at 40 weeks post-menstrual in good general condition, with a mild form of bronchopulmonary dysplasia (BPD) (grade 1 according to Jansen et al. [5]). He was readmitted to hospital at 43 weeks post-menstrual age due to thedevelopment of mixed stridor (inspiratory-expiratory) and worsening of respiratory symptoms, requiring the initiation high-flow oxygen therapy. Initially, we performed infection screening with blood culture (negative), urinalysis (negative), chest-x-ray and ultrasound (without evidence of anomalies), and multiplex reverse-transcriptase polymerase chain reaction (RT-PCR) on nasopharyngeal swabs for respiratory viruses and bacteria (including Mycoplasma pneumoniae, Chlamydophila pneumoniae, Legionella pneumoniae, and Bordetella pertussis)which were negative. Echocardiography was performed to assess cardiac patterns, and the results were normal. Due to the persistence of stridor, we performed an upper airway evaluation with direct laryngoscopy, which revealed type two laryngomalacia associated with mucosal oedema. After this evaluation, the patient’s clinical condition progressively worsened, with persistent stridor and an increased need for ventilatory support. In consultation with an otolaryngologist (ENT), we decided to perform laryngoplasty surgerythat discovered numerous SGCs which almost completely obstructed the airway (Fig. 1).

**Fig. 2:**
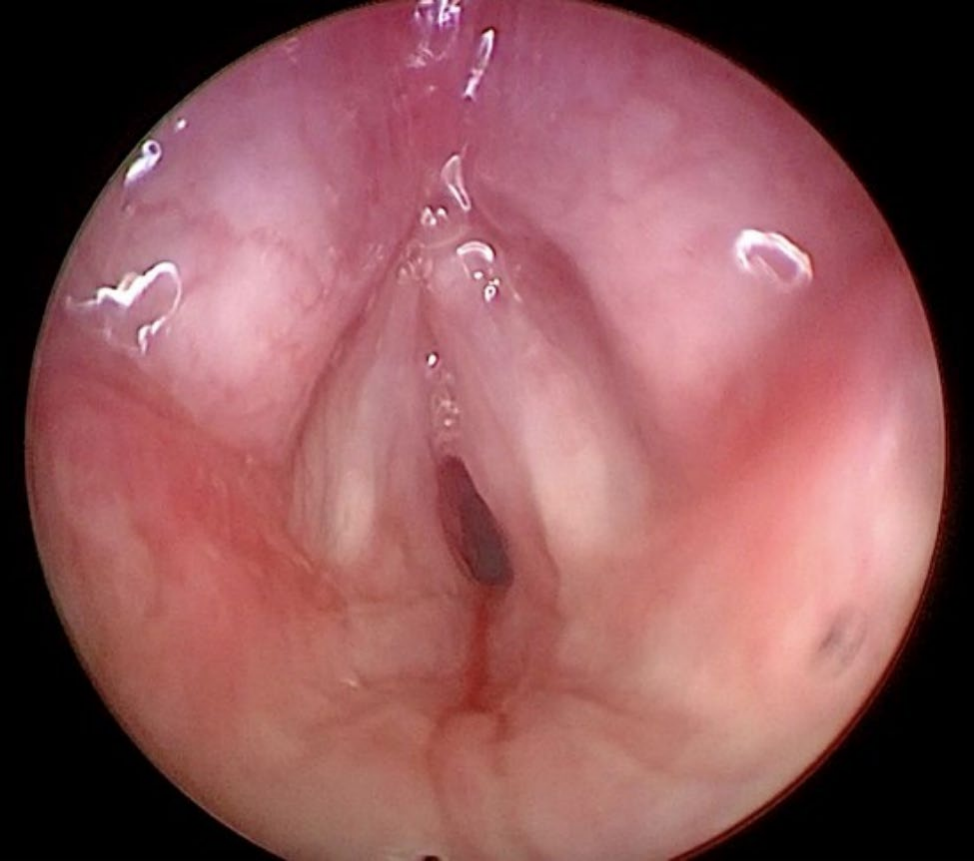
Post-operative control under endoscopic evaluation showing a better airway patency although a slight glottic edema persists

The ENT decided to remove the SGCs bycold excision method and performed a tracheostomy. A cuffed cannula of 2.5 mm diameter was positioned. No complications occurred during the positioning. There was a gradual improvement in respiratory condition. Post-surgery laringoscopy showed complete resolution with minimal oedema. (Fig. 2). Histopathological examination confirmed the benign nature of the lesion.

Initially, non-invasive ventilation support (CPAP) was placed. The next day, we assisted in the progressive improvement of clinical conditions, with the possibility of withdrawing ventilatory support after 8 days from surgery. Subsequent controls showed a completely patent airway and absence of SGCs. The tracheostomy tube was replaced twice during the hospital stay. The infant was discharged one monthafter surgery, with anti-reflux therapy. The tracheostomy was closed and left in placeSubsequent checks showed the presence of fibrous material associated with micro-cysts (maximum diameter 0.4 millimeters). Therefore, as a precaution, the endotracheal tube was not removed. 40 days after discharge, the tube is permanently removed.

## Discussion and conclusions

Our patient was an extremely premature male infant who required invasive mechanical ventilation on the first day of life. He had multiplerisk factors for the development of SGCs, including male sex, BPD, respiratory distress, prolonged intubation, and extreme prematurity [2]. Male infants appear to have the formation of SGC more frequently than female infants; this could be because there are more premature male infants than female infants [4]. Although the incidence is higher in patients who have been intubated, cases of subglottic cysts have also been reported in newborns who have never been intubated. This could suggest the existence of a congenital form, but due to early intubation in preterm infants, it is difficult to make a differential diagnosis between the two forms. The correlation between cyst formation and the duration of intubation is still a topic of debate in the literature. Smith et al. [6] showed that subglottic cysts can form after intubation for less than 24 h. On the other hand, Han et al. describe the direct correlation between the intubation period and the formation of SGCs [7]. The mechanism underlying the formation of these cysts is not yet known, and intubation can cause significant damage to the endotracheal mucosa, and this contributes to cyst formation. The frequent localization of the SGCs to the left side of the trachea and the subglottic area also supports the iatrogenic hypothesis [8, 9]. The left side of the trachea and subglottic area are usually affected by intubation by right-handed doctors, which supports the iatrogenic hypothesis [8]. Gastroesophageal reflux (GER) contributes to the formation of subglottic cysts and increases the risk of pathological scarring. The clinical presentation is often aspecific, as other acquired laryngeal stenoses can delay the diagnosis. The main clinical manifestations are the appearance of continuous biphasic stridor, obstructive apnea, respiratory distress, and feeding difficulties [9, 10]. These clinical signs, as in our case, can rapidly worsen and represent a danger to the patient’s life. Therefore, a complete evaluation of the upper airway should always be considered in preterm infants with a history of intubation. Laryngoscopy and bronchoscopy are the primary diagnostic tools [9]. Cyst marsupialization with cold steel instruments represents the technique mostly described in the literature; CO2 lasers and microdebriders are more prudently used because of the risk of thermal lesions on the surrounding mucosa [9, 10]. The recurrence of SGCs is frequent. Prolonged follow-up is mandatory in many cases, usually between 3 and 6 months after surgery, as a complementary endoscopic procedure may be required [11]. In conclusion, early diagnosis and treatment can positively influence the prognosis of these patients, avoiding the need for emergency life-saving treatments. In our case, due to the near-complete blockage of the airways by the cysts, a tracheostomy was necessary to ensure complete removal and maintain an open airway.

## Data Availability

the datasets during and/or analysed during the current study available from the corresponding author on reasonable request.

## Abbreviations

SGC: Subglottic cyst
GER: Gastro-esophageal reflux
HR: Heart rate
BPD: Bronchopulmonary dysplasia
RT-PCR: Multiplex reverse-transcriptase polymerase chain reaction
NICU: Neonatal Intensive Care Unit
ENT: Otolaryngologist
